# Zinc can counteract selection for ciprofloxacin resistance

**DOI:** 10.1093/femsle/fnaa038

**Published:** 2020-02-27

**Authors:** Michiel Vos, Louise Sibleyras, Lai Ka Lo, Elze Hesse, William Gaze, Uli Klümper

**Affiliations:** 1 European Centre for Environment and Human Health, University of Exeter Medical School, Knowledge Spa, Royal Cornwall Hospital Truro, TR1 3HDTruro, Cornwall, UK; 2 Université Paris Saclay, Department of Biology, Espace Technologique Bat. Discovery - RD 128 - 2e ét, 91190 Saint-Aubin, France; 3 Institute for Evolution & Biodiversity, Universität Münster, Hüfferstraße 1, 48149 Münster, Germany; 4 College of Life and Environmental Science, University of Exeter, Penryn Campus, TR10 9FE Penryn, Cornwall, UK; 5 Environment and Sustainability Institute, University of Exeter, Penryn Campus, TR10 9FE Penryn, Cornwall, UK

**Keywords:** Antimicrobial resistance, Selection dynamics, Heavy metals, Chelation, Fluroquinolone, Antibiotic resistance

## Abstract

Antimicrobial resistance (AMR) has emerged as one of the most pressing threats to public health. AMR evolution occurs in the clinic but also in the environment, where antibiotics and heavy metals can select and co-select for AMR. While the selective potential of both antibiotics and metals is increasingly well-characterized, experimental studies exploring their combined effects on AMR evolution are rare. It has previously been demonstrated that fluoroquinolone antibiotics such as ciprofloxacin can chelate metal ions. To investigate how ciprofloxacin resistance is affected by the presence of metals, we quantified selection dynamics between a ciprofloxacin-susceptible and a ciprofloxacin-resistant *Escherichia coli* strain across a gradient of ciprofloxacin concentrations in presence and absence of zinc. The presence of zinc reduced growth of both strains, while ciprofloxacin inhibited exclusively the susceptible one. When present in combination zinc retained its inhibitory effect, while ciprofloxacin inhibition of the susceptible strain was reduced. Consequently, the minimal selective concentration for ciprofloxacin resistance increased up to five-fold in the presence of zinc. Environmental pollution usually comprises complex mixtures of antimicrobial agents. In addition to the usual focus on additive or synergistic interactions in complex selective mixtures, our findings highlight the importance of antagonistic selective interactions when considering resistance evolution.

## Teaser

Selection for ciprofloxacin resistance in a bacterial strain is reduced when zinc cations are simultaneously present during exposure to the antibiotic ciprofloxacin.

## INTRODUCTION

The emergence and spread of antimicrobial resistance (AMR) genes in bacterial pathogens constitutes a major threat to human health (WHO 2014). Although AMR genes are ancient and have evolved as a result of microbial interactions, as for example evidenced by their presence in permafrost samples minimally impacted by anthropogenic activity (D'Costa *et al*. 2011; Perron *et al*. 2015), the use and misuse of antibiotics in healthcare and agriculture has caused a rapid and worrying increase in the prevalence of AMR in the human as well as environmental microbiomes (Knapp *et al*. 2010). The environmental dimensions of AMR evolution are increasingly appreciated (Wellington *et al*. 2013; Larsson *et al*. 2018; Smalla *et al*. 2018), with two major research strands emerging relating to selection for resistance. First, recent studies utilising both single species (Gullberg et al. 2011, 2014; Liu *et al*. 2011; Klümper *et al*. 2019b) and complex microbial communities (Lundström *et al*. 2016; Kraupner *et al*. 2018; Murray *et al*. 2018) have demonstrated that selection for AMR can occur at antibiotic concentrations much lower than those preventing the growth of susceptible bacteria. These studies highlight the importance of considering the minimal selective concentration (MSC) in addition to the minimal inhibitory concentration (MIC) for assessing risks associated with antibiotic concentrations in the environment. Second, environmental pollution with non-antibiotic compounds such as biocides and/or metals can contribute to the spread and selection of AMR through processes such as cross-resistance, co-selection and co-regulation (Baker-Austin *et al*. 2006; Pal *et al*. 2017; Dickinson *et al*. 2019) or by altering transfer dynamics of AMR plasmids (Klümper *et al*. 2017, 2019a). Pollution with metals is especially problematic as metals are highly persistent and toxic even at low concentrations (Gadd and Griffiths 1977). In certain environmental settings, heavy metals such as copper (Cu) and zinc (Zn) may even constitute stronger selective agents for antibiotic resistance than antibiotics (Song *et al*. 2017).

While the selective potential of antibiotics and heavy metals for AMR has been well-characterized, experimental studies exploring their combined effect on resistance evolution are relatively rare. The presence of a second antibiotic could, for example, either potentiate or decrease antibiotic efficacy (Cao *et al*. 2012; Churski *et al*. 2012) and hence cause a decrease or increase in MSC. Metals and antibiotics can similarly have synergistic or antagonistic effects. For example, metal complexation can decrease the hydrolysis potential of *β*-lactam antibiotics by *β*-lactamases and hence increase antibiotic potency (Anacona 2001). Additionally, the selective potential of mechanisms conferring co-resistance to metals and antibiotics, such as efflux pumps, can be increased in the presence of metals (Mata, Baquero and Pérez-Díaz 2000; Aendekerk *et al*. 2002). Lastly, it is also possible that metals could diminish the activity of antibiotics by binding and inactivation (Li, Nix and Schentag 1994). As metal-antibiotic interactions are varied and highly relevant in environmental pollution scenarios, it is important to address how antibiotics and metals jointly affect bacterial resistance spread.

Fluoroquinolones are recognized as critically important antibiotics for human health by the WHO (WHO 2016) and are characterized by a high degree of persistence in the environment (Kümmerer, Al-Ahmad and Mersch-Sundermann 2000). Concentrations in environmental settings range from low ng/L to μg/L, while exceptionally high levels of ciprofloxacin in the mg/L range have been found in effluents from drug manufacturers and in nearby industrially polluted environments (Larsson, de Pedro and Paxeus 2007; Fick *et al*. 2009; Gothwal and Shashidhar 2015). Interactions between fluoroquinolones and metals have received previous attention but with mixed results. In metal(II)-ciprofloxacin complexes, the drug ligand is coordinated through two carbonyl oxygen atoms (Chohan, Supuran and Scozzafava 2005). Among these, Zn(II)-ciprofloxacin complexes were shown to have better solubility and greater activity against Gram-positive and Gram-negative pathogens compared to uncomplexed ciprofloxacin (Chohan, Supuran and Scozzafava 2005; Imran *et al*. 2007; Patel, Chhasatia and Parmar 2010). However, a number of studies have also demonstrated that metal-chelated ciprofloxacin, even whilst more readily transported across the bacterial cell membrane, has reduced antimicrobial efficacy (Li, Nix and Schentag 1994; Ma, Chiu and Li 1997; Seedher and Agarwal 2010).

To test whether metal chelation could impact selection for antibiotic resistance, we here quantified growth rates of a ciprofloxacin-susceptible and a ciprofloxacin-resistant *Escherichia coli* MG1655 strain across a gradient of ciprofloxacin concentrations in the presence and absence of Zn. We also performed a competition experiment between both strains in mine-waste contaminated stream water to test whether complex environmental mixtures of metals could affect selection for ciprofloxacin resistance. Our results shed light on a hitherto unappreciated dimension of environmental AMR selection, namely, that there is a selective window where metal pollution may reduce the selective effects of fluoroquinolone antibiotics.

## MATERIAL AND METHODS

### Strains

An *Escherichia coli* MG1655 strain was chromosomally tagged with a Tn*7* gene cassette encoding constitutive red fluorescence, expressed by the *mCherry* gene (Klümper, Dechesne and Smets 2014; Klümper *et al*. 2015). A strain resistant to ciprofloxacin and a strain resistant to gentamicin were derived from this red fluorescent strain. The ciprofloxacin resistant strain was created through spontaneous chromosomal mutation by evolving the susceptible ancestor by serial inoculation of overnight culture in LB medium (10 g/L Tryptone, 10 g/L NaCl and 5 g/L Yeast Extract) supplemented with incremental concentrations of ciprofloxacin (0.0625, 0.125, 0.25, 0.5 and 1 µg/mL), until a resistant phenotype evolved able to grow at the highest concentration. A gentamicin resistant strain was constructed through electroporation of the susceptible ancestor with the pBAM delivery plasmid containing the mini-Tn*5* delivery system (Martínez-García *et al*. 2011) for gentamicin resistance gene *aacC1* encoding a gentamicin 3′-N-acetyltransferase (Kovach *et al*. 1995). The ciprofloxacin- and gentamicin-resistant strains as well as the ancestral susceptible strain were grown overnight in sterile LB broth (supplemented with 0.5 μg/mL ciprofloxacin, 10 μg/mL gentamicin or no antibiotics, respectively), harvested by centrifugation (3500 × *g*, 20 min, 4°C), three times washed with sterile 0.9% NaCl solution to remove residual antibiotics with the finally density adjusted to OD_600_ 0.1 (∼10^7^ bacteria/mL) in sterile 0.9% NaCl solution for use in experiments. All chemicals used in this study were supplied by Sigma-Aldrich (St. Louis, MO, USA).

### Water sampling

A water sample was collected in May 2019 from the Carnon River near Bissoe, Cornwall, UK (50°13’54.6’ N, 5°07’48.7’ W). The surrounding area has a long history of mining-related heavy metal contamination (Pirrie *et al*. 2003; Environment Agency UK 2019), resulting in contamination of soils, sediments and water with a complex mixture of metals, including zinc. A sterile 1 L Duran bottle was filled and immediately transported back to the laboratory to use for the growth rate and competition assays on the same day of sampling. The water was supplemented with sterile LB broth powder (25 g/L) and sterilized through 0.2 μm^2^ pore filters (Whatman). Sterility was confirmed by incubating 10 mL of the liquid medium at 37°C overnight. An aliquot of the water sample was frozen at -20åC and analysed in triplicate for metal content using ICP-MS on an Agilent 7700x (Agilent Technologies, Santa Clara, Ca, USA) at the Camborne School of Mines laboratory at the University of Exeter (Table S1, Supporting Information).

### Growth rate assays

Individual strains, originating from a single overnight culture, were inoculated in 96 well plates at ∼10^5^ cells/mL in technical triplicates into 300 μL LB broth supplemented with ZnSO_4_ (0.0, 0.5 and 1 mM) and either ciprofloxacin (11 two-fold decreasing concentrations starting at 0.4 μg/mL) or gentamicin (11 two-fold decreasing concentrations starting at 10 μg/mL). To account for different batches of LB broth as a complex growth medium potentially containing minimal background levels of Zn as a micronutrient, all experiments were carried out using LB from the same 1 kg batch. After 24 h growth under shaking conditions (120 rpm) OD_600_ was measured to determine bacterial growth using a Synergy 2 spectrophotometer (Biotek Instruments Inc., VT, USA). Individual growth rates of resistant strains were calculated relative to growth of the susceptible strain in a control treatment unamended with zinc or antibiotics.

Relative fitness of the susceptible strain (*ρ_s_*) was calculated using the specific growth rate (*γ*) of the susceptible (s) and the resistant (r) strain at each individual concentration, with bacterial numbers (n) estimated using OD_600_ measurements at time 0 (T_0h_) and after 24 h (T_24h_).

$$\begin{equation*}
{\rho _s}\ = \frac{{{\gamma _s}}}{{{\gamma _r}}}\ = \ \frac{{\ln \Big({{{n_s^{{T_{24h}}}}} \!/ \!{{n_s^{{T_{0h}}}}}}\Big)}}{{\ln \Big({{{n_r^{{T_{24h}}}}} \!/ \!{{n_r^{{T_{0h}}}}}}\Big)}}
\end{equation*}$$

The experimental data linking relative fitness to antibiotic exposure at the different metal doses were fitted with a four parameter log-logistic dose-response curve using the ‘drc’ (analysis of dose-response curves) package in R (Knezevic, Streibig and Ritz 2007) with separate curves fitted for each level of zinc, using ‘curveid’ with Zn as factor. To integrate the no antibiotic control (c = 0 μg/mL) in the log-logistic model its concentration was set to a minimum threshold as per the developers’ instructions (i.e. by dividing lowest non-zero value by 10). Using these modelled curves the MSC at which *ρ*_s_ = *ρ*_r _= 1 was calculated for each scenario, as well as their standard deviation and 95% confidence intervals. To test if Zn alters the dose response relationship, the fitted model was compared to a reduced model where fitness data were pooled across ciprofloxacin concentrations.

### Competition assay

The gentamicin-resistant, ciprofloxacin-susceptible strain was competed with the gentamicin-susceptible, ciprofloxacin-resistant strain. This allowed for simple identification of the ciprofloxacin-susceptible and resistant strain on selective plates. The pair was inoculated at a 1:1 ratio and initial density of ∼10^5^ cells/mL into 10 mL of LB broth supplemented with ZnSO_4_ (0.0 and 0.5 mM) and ciprofloxacin (0.001, 0.01, 0.025 and 0.1 μg/mL) in triplicate 30 mL glass vials. Glass vials were incubated shaken (120 rpm) at 37°C for 24 h, after which 100 μL (1%) was transferred daily to fresh LB broth for a total of seven days. To obtain initial (T_0d_) and final (T_7d_) cell densities for each vial, dilution series were prepared in sterile 0.9% NaCl solution and plated on LB agar containing either 10 μg/mL gentamicin (selective for ciprofloxacin-susceptible strain) or 0.5 μg/mL ciprofloxacin (selective for ciprofloxacin-resistant strain). Plating of the respective strains on the counter selecting plates did not lead to any growth of spontaneous mutants. The relative fitness of the susceptible strain was calculated as before using growth rate ratios.

### Statistics

For each experimental condition (combination of Zn and ciprofloxacin concentration), we used a two-tailed *t*-test to test whether the relative fitness of the susceptible strain differed from a scenario with no selection (*ρ* = 1). We used a similar approach to test whether under a given experimental condition relative growth of either strain significantly differed from that of the susceptible one in the absence of antibiotics and zinc. To compare the relative fitness or growth rates between different experimental conditions ANOVA tests were performed.

## RESULTS

### Effect of Zinc on selection for ciprofloxacin resistance

**Figure 1.**
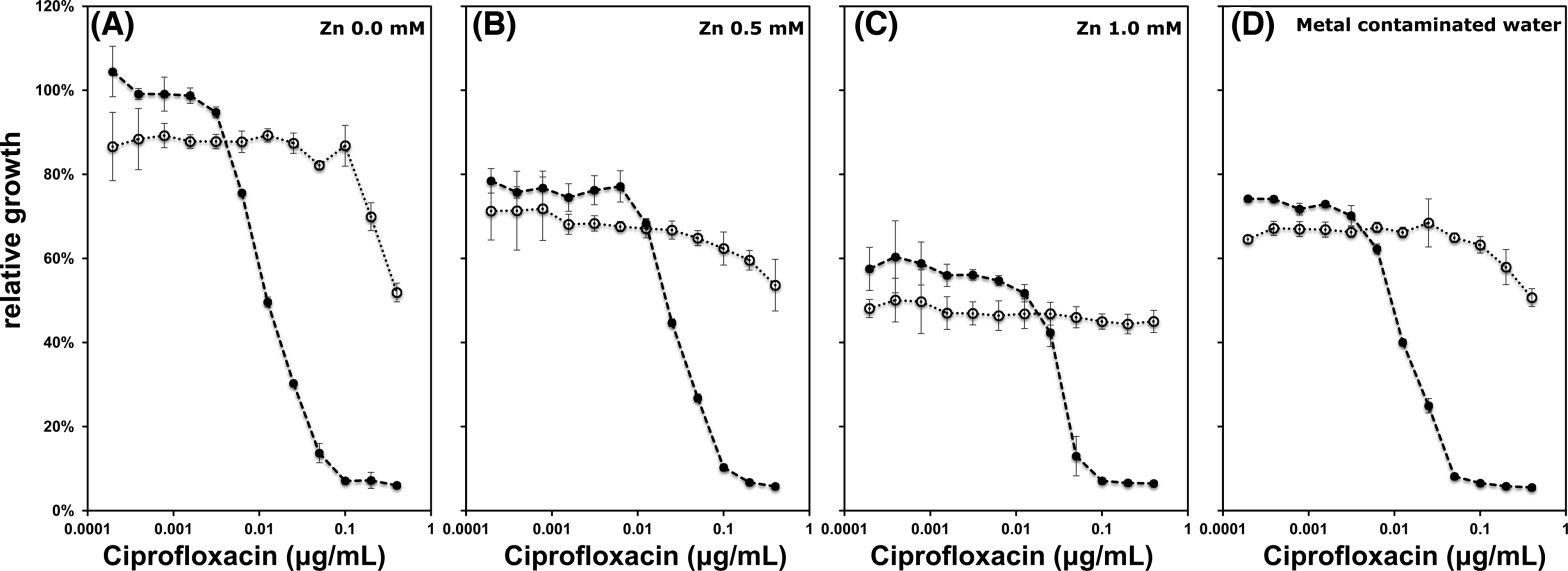
Growth rate of a ciprofloxacin-susceptible strain (filled circles) and a ciprofloxacin-resistant strain (open circles) in LB medium amended with ciprofloxacin and zinc as well as in metal contaminated water supplemented with LB, relative to the growth rate of the ciprofloxacin-susceptible strain in control LB. Growth was quantified based on OD_600_ after 24 h of growth at 37åC (n = 3, average ± SD).

To determine the effect of zinc on selection for ciprofloxacin resistance, ciprofloxacin-resistant and ciprofloxacin-susceptible *E. coli* strains were individually grown across a gradient of ciprofloxacin and Zn concentrations for 24 h. The growth rate of each strain was plotted relative to that of the ciprofloxacin-susceptible strain grown in control LB (without ciprofloxacin and Zn) (Fig. 1A-C; Table S2, Supporting Information). Growth of both strains significantly decreased with increasing Zn concentrations in the absence of ciprofloxacin to 78.7 ± 4.1% (0.5 mM, *P *< 0.0001, two-tailed *t*-test against 1) and 55.4 ± 2.4% (1 mM, *P *< 0.0001, two-tailed *t*-test against 1). Ciprofloxacin resistance caused a small but significant reduction in relative growth rate in the absence of antibiotics and metals (82.8 ± 3.4% relative growth, mean ± SD) (*P *= 0.012, two-tailed *t*-test against 1) (Fig. 1A). This cost of resistance was also apparent across both Zn concentrations tested (0.5 mM: 90.7 ± 5.3%, *P *= 0.093; 1 mM: 83.8 ± 3.7%, *P *= 0.0161, two-tailed *t*-test against 1) (Fig. 1B and C).

**Figure 2.**
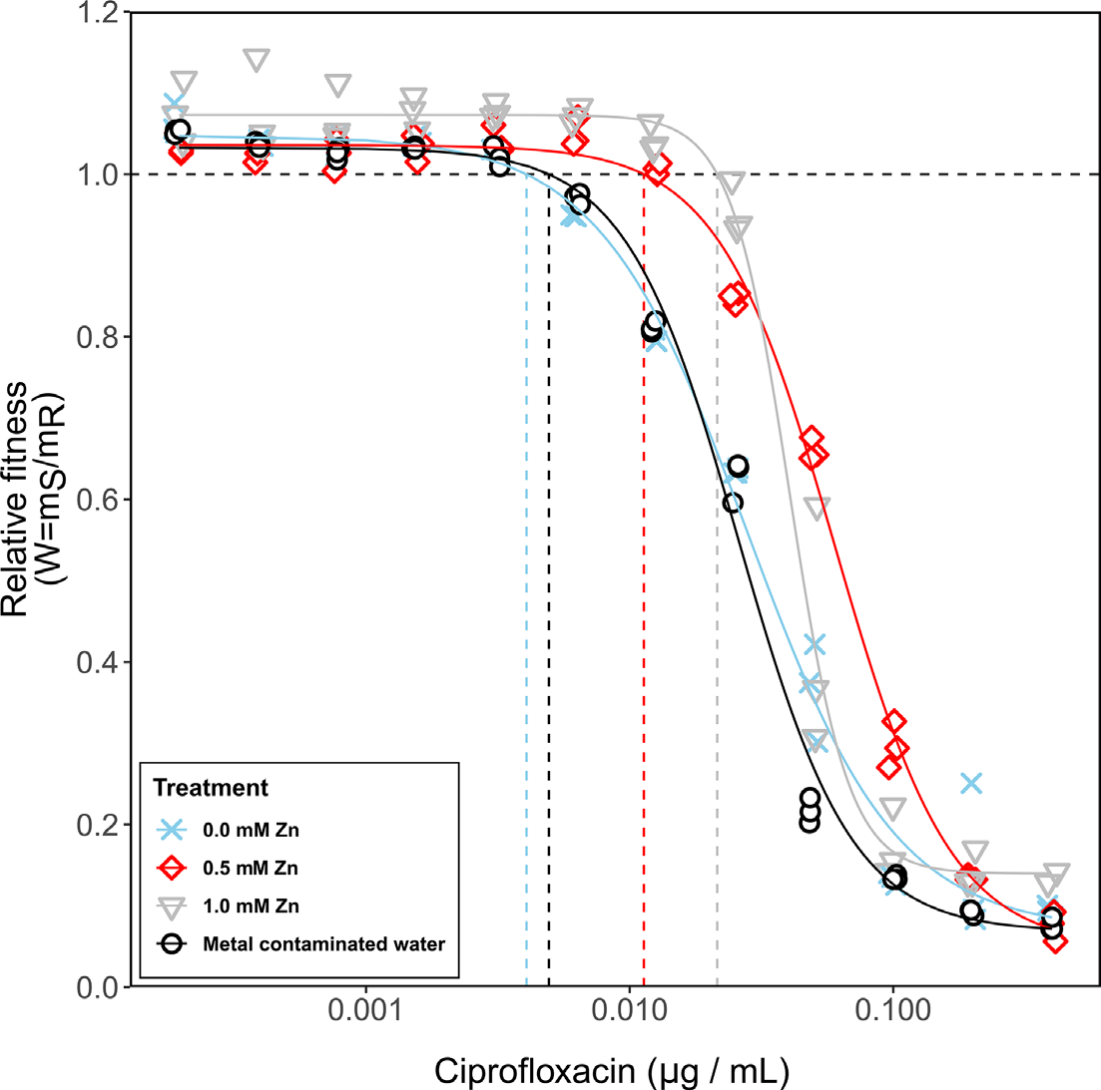
Fitness of a ciprofloxacin susceptible *E. coli* strain relative to fitness of an isogenic ciprofloxacin resistant strain across a gradient of ciprofloxacin for three LB broth treatments amended with Zn and one metal contaminated water source supplemented with LB. Horizontal dashed line indicates different strains perform equally well (*ρ*_s_ = *ρ*_r_ = 1), and the vertical lines represent the corresponding minimal selective concentration for the different Zn treatments.

A significant growth rate advantage for the ciprofloxacin-resistant strain was detected at 0.00625 μg/mL ciprofloxacin (*ρ*_s,0 mM _= 0.949 ± 0.001, *P *= 0.0002, two-tailed *t*-test against 1) in the absence of Zn (Fig. 2). However, at the same antibiotic concentration in the presence of both Zn concentrations, this advantage disappeared (0.5 mM: *ρ*_s _= 1.050 ± 0.018, *P *= 0.0412; 1.0 mM: *ρ*_s,1.0 mM _= 1.073 ± 0.010, *P *= 0.0289; two-tailed *t*-test against 1). In the presence of 0.5 mM Zn, the minimal concentration with a significant selective advantage for the resistant strain increased from 0.00625 μg/mL ciprofloxacin to 0.025 μg/mL (*ρ*_s _= 0.848 ± 0.008; *P *= 0.0008, two-tailed *t*-test against 1) and for 1 mM Zn to 0.05 μg/mL (*ρ*_s _= 0.422 ± 0.150; *P *= 0.0218, two-tailed *t*-test against 1) (Fig. 2).

We fitted a log-logistic dose response model to the relative fitness values across the antibiotic gradient for different Zn concentrations (Fig. 2). This significantly improved the fit compared to a reduced model (F_12,140 _= 51.113, *P *< 0.001, ANOVA), suggesting that increasing Zn concentrations alter the dose response relationship. Based on the full model, MSCs were estimated as the intercept with ρ_s _= 1 (no selection) and significantly increased from 0.0041 ± 0.0005 μg/mL in the absence of Zn to 0.0113 ± 0.0013 μg/mL (0.5 mM Zn) and further to 0.0215 ± 0.0014 μg/mL (1.0 mM Zn) (Table 1).

**Table 1.** Minimal selective concentrations (MSC) of ciprofloxacin modelled for the different Zn^2+^ concentrations and metal contaminated water.

Medium	MSC	Lower 95% CI	Upper 95% CI
0.0 mM Zn^2+^	0.0041 ± 0.0005 μg/mL	0.0030 μg/mL	0.0051 μg/mL
0.5 mM Zn^2+^	0.0113 ± 0.0013 μg/mL	0.0087 μg/mL	0.0139 μg/mL
1.0 mM Zn^2+^	0.0215 ± 0.0014 μg/mL	0.0188 μg/mL	0.0242 μg/mL
Metal contaminated water	0.0049 ± 0.0006 μg/mL	0.0037 μg/mL	0.0062 μg/mL

Zn also affected the relative growth rate of the ciprofloxacin-resistant strain: growth significantly decreased at 0.2 and 0.4 μg/mL ciprofloxacin (*P *= 0.002–0.0498, df = 5, ANOVA) for no Zn and 0.5 mM of Zn (Fig. 1A and B). No such decrease in growth was detected when exposed to 1 mM of Zn (*P *= 0.114–0.194, df = 5, ANOVA) (Fig. 1C). Selection dynamics for gentamicin, a non-fluoroquinolone antibiotic that does not chelate metals, were not similarly affected by Zn (Figs S1 and S2, Supporting Information).

### Effect of environmental metal contamination on selection for ciprofloxacin resistance

To explore the relevance of the results under more natural conditions, we performed a competition experiment for ciprofloxacin using sterilized water from the metal-contaminated Carnon River (Cornwall, United Kingdom; Table S1, Supporting Information) amended with LB. The contaminated water significantly decreased relative growth of both the ciprofloxacin-resistant (77.9 ± 1.0%, *P *= 0.0007, two-tailed *t*-test against 1) and ciprofloxacin-susceptible strain (74.2 ± 0.6% *P *= 0.0002, two-tailed *t*-test against 1) relative to control medium made up with deionized water. In the absence of ciprofloxacin the susceptible strain had greater fitness relative to the resistant one (*ρ*_s _= 1.052 ± 0.003; *P *= 0.0012, two-tailed *t*-test against 1). Growth inhibition (22.1 ± 1.0%) was quantitatively and qualitatively similar to that observed for 0.5 mM Zn (21.3 ± 4.1% *P *= 0.426, df = 10, ANOVA) (Fig. 1D). However, the MSC estimated from the dose response curve model was at 0.0049 ± 0.0006 μg/mL ciprofloxacin, similar to that observed for non Zn-amended broth (Table 1). Again a significant decrease in growth rate of the resistant strain at high concentrations of 0.4 μg/mL ciprofloxacin was observed in naturally metal contaminated water (*P *= 0.0005, df = 5, ANOVA) (Fig. 1D).

**Figure 3.**
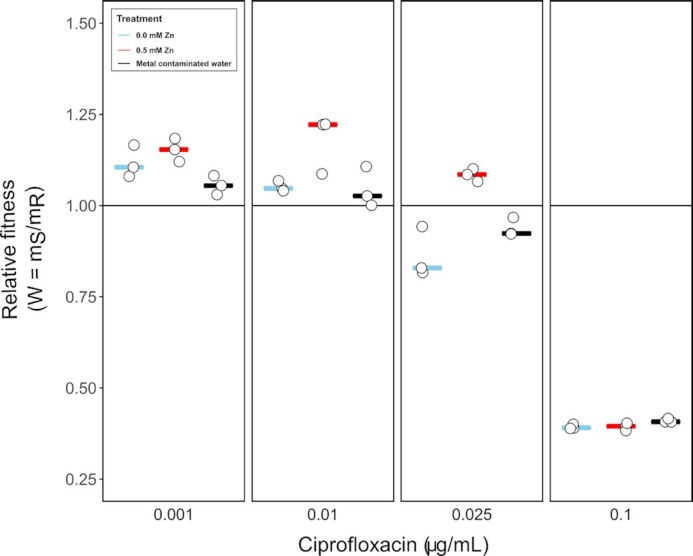
Relative fitness of the ciprofloxacin-susceptible strain based on seven-day competition experiments across a gradient of ciprofloxacin in the absence and presence of 0.5 mM Zn and in naturally metal contaminated water.

To test whether differences in growth rate translate to selection dynamics, the ciprofloxacin-susceptible and ciprofloxacin-resistant strains were competed for seven days across four ciprofloxacin concentrations in LB Broth in the presence and absence of 0.5 mM Zn as well as in naturally metal contaminated water (Fig. 3). Again, relative fitness of the susceptible strain did not significantly differ between the unpolluted control and metal contaminated water at any of the tested ciprofloxacin concentrations (all *P *> 0.05, ANOVA). While metal contaminated water had an effect on the growth rates of both susceptible and resistant strains (Fig. 1A and D), no effect on selection for ciprofloxacin resistance was observed in the environmental sample. This is most likely explained by the fact that the total metal content was high enough to negatively affect growth, but that the concentration of Zn (0.028 mM; Table S1, Supporting Information) and other chelating metals was too low to significantly affect ciprofloxacin. The seven-day selection experiment supported the results of the short-term growth assays in that it showed the competitive advantage of a ciprofloxacin-susceptible strain in the presence of Zn. This is evidenced by the fact that at 0.5 mM Zn, the susceptible strain outcompetes the resistant strain at 0.025 μg/mL ciprofloxacin (*ρ*_s,0.5 mM _= 1.084 ± 0.017), whereas the reverse is true in the absence of Zn (*ρ*_s,0.0 mM _= 0.863 ± 0.069; *P *= 0.0058, df = 5, ANOVA) (Fig. 3). The effect of Zn is apparent at concentrations as low as 0.01 μg/mL ciprofloxacin (*ρ*_s,0.5 mM _= 1.177 ± 0.078; *ρ*_s,0.0 mM _= 1.052 ± 0.014; *P *= 0.053, df = 5, ANOVA) (Fig. 3).

## DISCUSSION

Here, we present evidence that metals have the potential to reduce selection for ciprofloxacin resistance. The addition of zinc resulted in a five-fold increase in the MSC for ciprofloxacin resistance at a concentration of 1.0 mM Zn. Zinc was found to reduce both absolute growth rate and final density of the ciprofloxacin-resistant strain, hence decreasing the total incidence of resistant phenotypes. The non-fluoroquinolone antibiotic gentamicin, which does not chelate metal, did not exhibit a similar interaction with Zn, which is consistent with a scenario where zinc reduces selection for ciprofloxacin resistance through metal-antibiotic specific formation of chelates.

Fluoroquinolones, such as ciprofloxacin, are hydrophilic and hence possess high mobility in aquatic environments (Felis *et al*. 2020). In effluents from hospitals, concentrations of ciprofloxacin reach levels as high as 0.026 μg/mL (Verlicchi *et al*. 2012), well in the range of effect concentrations determined in this study. Despite its relatively low stability with half-life times estimated as approximately 46 hours (Cardoza *et al*. 2005; Felis *et al*. 2020), ciprofloxacin has regularly been detected in diverse aquatic environments such as groundwater and drinking water (Hanna *et al*. 2018; Reis *et al*. 2019).

Ciprofloxacin resistance in *E. coli* can be conferred through diverse genetic changes which consequently confer diverse fitness costs/gains on the ciprofloxacin resistant isolates (Marcusson, Frimodt-Møller and Hughes 2009; Baker *et al*. 2013; Machuca *et al*. 2014). Most strains unable to develop a favourable set of mutations in the quinolone resistance-determining regions (QRDR) have to rely primarily on the overexpression of efflux pumps which is energetically demanding and impacts fitness (Johnson *et al*. 2015; Fuzi, Szabo and Csercsik 2017). Only a few *E. coli* Sequence Types (ST131, ST1193) are capable of evolving multiple energetically favourable QRDR mutations in the gyrase or topoisomerase IV (Fuzi, Szabo and Csercsik 2017), which might have contributed to their widespread dissemination. In our study, ciprofloxacin resistance conferred a major fitness cost on *E. coli* strain MG1655. This resulted in an increased MSC in the presence of Zn, mainly by decreasing the inhibitory effects of ciprofloxacin on the susceptible strain. For strains with neutral or favourable QRDR mutations selection for resistance is positive or neutral at any concentration, thus no direct effect on the MSC is to be expected. However, the Zn induced decrease in ciprofloxacin inhibition of the susceptible strain remains relevant in these scenarios as it will allow susceptible strains to persist as competitors at an increased range of ciprofloxacin concentrations.

The effect of Zn on selection for ciprofloxacin resistance was apparent at relatively high metal concentrations (0.5–1 mM) in artificial media. Experiments performed using a broth-supplemented metal-contaminated water source did not reveal an effect on selection dynamics. This can be explained by the fact that the environmental Zn concentration was over an order of magnitude lower (0.028 mM, Table S1, Supporting Information) than the lowest defined concentration used (even though the environmental sample contained a range of other metals implicated in fluoroquinolone chelation (Ma, Chiu and Li 1997)). However, it remains possible that other, more heavily polluted sites have metal concentrations high enough to interfere with selection for resistance to low levels of fluoroquinolone. For example, landfill leachates have been shown to contain Zn concentrations reaching up to 3.8 mM (Roy 1994), higher than those concentrations we have demonstrated affecting selection for ciprofloxacin resistance.

Our results could have implications for human or veterinary medicine. For instance, prevalence of ciprofloxacin resistance can reach up to 21.2% of all *Enterococci* spp. isolates in pig manure samples (Hölzel *et al*. 2010), making any factors altering selection dynamics highly relevant. Zinc compounds are regularly used as agricultural feed additives and growth promoters in agriculture (Poulsen 1998; Castillo *et al*. 2008) with concentrations in liquid pig manure reaching up to 4 mmol/kg wet weight (Hölzel *et al*. 2012), considerably higher than the concentrations used here. In humans, where ciprofloxacin is prescribed for a wide range of infections caused by both Gram-negative and Gram-positive bacteria (Redgrave *et al*. 2014), the effects observed here could potentially be relevant when patients take zinc supplements, for which daily doses of as high as 40 mg have been reported (Liu *et al*. 2017). It remains, however, crucial to take speciation and bioavailability rather than total Zn measurements into account.

The focus of this study was exclusively on selection for pre-existing antibiotic resistance mutations in a focal species. Co-occurrence of metal and antibiotic stressors could, however, have additional effects on *de novo* evolution of antibiotic resistance. Metal stress could for example increase the rate at which antibiotic and metal resistance evolves through increased mutation rate (Lemire, Harrison and Turner 2013). In contrast, effects of metals on the spread of antibiotic resistance might be harder to predict when embedded in a complex microbial community. Selection for resistance has been shown to be further reduced when competing with other community members (Klümper *et al*. 2019b). However, in complex communities a greater source of resistance genes will be available to the focal species through horizontal gene transfer. While chromosomal resistance determinants could be lost through negative selection, those embedded on self-transmissible plasmids can persist or even increase in abundance, as a consequence of their sometimes extremely broad host ranges and high transfer frequencies (Musovic *et al*. 2014; Shintani *et al*. 2014; Klümper *et al*. 2015; Arias-Andres *et al*. 2018). Transfer rates of resistance plasmids can consequently be directly impacted by metals, both positively or negatively (Klümper et al. 2017, 2019a).

In summary, we have shown that a selective window exists in which zinc reduces the strength of selection on ciprofloxacin resistance under laboratory conditions. It is highly likely that additional metal-antibiotic interactions affect the efficacy of antibiotics (Li, Nix and Schentag 1994), and hence these deserve future research attention. Our data highlight that co-occurring metals and antibiotic residues add a further level of complexity when assessing the risks of environmental AMR evolution, but it remains to be tested whether these conditions occur in real-world pollution scenarios.

## Supplementary Material

fnaa038_Supplemental_File
