# Supplementary material for: Zinc can counteract selection for ciprofloxacin resistance
Source: FEMS Microbiol Lett. 2020 Feb 27;367(3):fnaa038. doi: 10.1093/femsle/fnaa038 (PMC7082703; doi:10.1093/femsle/fnaa038)
Supplement: fnaa038_Supplemental_File [file fnaa038_supplemental_file.docx]

## Supplementary information

## Title

## Zinc can counteract selection for ciprofloxacin resistance

## Author list

Michiel Vos ^1^, Louise Sibleyras ^1,2^, Lai Ka Lo ^1,3,4^, Elze Hesse ^4,5^, William Gaze ^1^, Uli Klümper ^1,4,5*^

^1^ European Centre for Environment and Human Health, University of Exeter Medical School, Truro, Cornwall, United Kingdom

^2^ Université Paris Saclay, Paris, France

^3^ Institute for Evolution & Biodiversity, Universität Münster, Germany

^4^ College of Life and Environmental Science, University of Exeter, Penryn, Cornwall, United Kingdom

^5^ Environment and Sustainability Institute, University of Exeter, Penryn, Cornwall, United Kingdom

^*^corresponding author:

Uli Klümper

CLES & ESI University of Exeter

TR109FE Penryn

United Kingdom

Email: [u.klumper@exeter.ac.uk](mailto:u.klumper@exeter.ac.uk)

Phone: (+44)7497497338

ORCID: 0000-0002-4169-6548

**Content:**

**SI Text: Effect of Zinc on selection for gentamicin resistance**

**SI Table 1: Metal concentrations in metal contaminated water** from the Carnon river near Bissoe, Cornwall, UK measured by ICP-MS

**SI Table 2: Relative growth of the susceptible and the ciprofloxacin resistant strain across a gradient of ciprofloxacin and zinc concentrations.** Growth was quantified based on OD_600_ after 24 h of growth at 37˚C (n=3, average ± SD) compared to the susceptible strain in the absence of antibiotic or metal.

**SI Figure 1: Relative growth of the susceptible (filled circles) and the gentamicin resistant (open circles) strain across a gradient of gentamicin and zinc concentrations.** Growth was quantified based on OD_600_ after 24 h of growth at 37˚C (n=3, average ± SD) compared to the susceptible strain in the absence of antibiotic or metal.

**SI Figure 2: Relative fitness of the gentamicin susceptible strain across a gradient of gentamicin and zinc concentrations.** Horizontal dashed line indicates no selection at a relative fitness of *ρ*_s_ = *ρ*_r_ = 1. The intercept with the reduced log-logistic dose response model indicates the minimal selective concentration illustrated with a vertical dashed line. Since no significant influence of Zn on the log-logistic dose response model was detected exclusively the reduced models selection curve with MSC for gentamicin at 0.0242 ± 0.0162 μg/mL is shown.

**SI Text:**

**Effect of Zinc on selection for gentamicin resistance**

We repeated the experiment described above with gentamicin as the focal antibiotic. Isogenic *E. coli* strains with and without gentamicin resistance were grown in isolation across a gradient of gentamicin and Zn concentrations for 24h. Growth of the gentamicin-resistant strain was reduced to 90.3 ± 1.3% relative to its susceptible counterpart in the absence of antibiotics and metals (p=0.0001, two-tailed *t*-test against 1). This cost of resistance was also apparent in the presence of Zn (0.5mM: 90.7 ± 1.0%, p=0.0001; 1.0 mM: 90.4 ± 3.6%, p=0.0005; ANOVA) (Figure SI1). Increasing Zn concentrations significantly decreased growth of both the resistant and sensitive strains in the absence of gentamicin (18.7 ± 1.0% at 0.5 mM, p<0.0001; 46.6 ± 1.4% at 1 mM, p<0.0001; ANOVA). However, unlike ciprofloxacin, the dose response curves estimated for the different Zn concentrations were not significantly different (model compared to reduced model: F_8,104_ =0.90, p=0.52; Figure SI2).

**SI Table 1: Metal concentrations in metal contaminated water** from the Carnon river near Bissoe, Cornwall, UK measured by ICP-MS

| **Metal** | **Concentration** |  | **Molarity** |
| --- | --- | --- | --- |
| **Mn** | 443.20 ± 11.99 ng/mL |  | 8.058 ± 0.218 μM |
| **Fe** | 2.50 ± 0.43 ng/mL |  | 0.045 ± 0.008 μM |
| **Ni** | 60.44 ± 3.90 ng/mL |  | 1.007 ± 0.065 μM |
| **Cu** | 439.19 ± 3.98 ng/mL |  | 6.971 ± 0.063 μM |
| **Zn** | 1837.40 ± 21.12 ng/mL |  | 27.839 ± 0.320 μM |
| **As** | 0.32 ± 0.02 ng/mL |  | 0.004 ± 0.000 μM |
| **Cd** | 2.52 ± 0.14 ng/mL |  | 0.023 ± 0.001 μM |
| **Pb** | 0.05 ± 0.01 ng/mL |  | 0.000 ± 0.000 μM |

| **Strain** | **Ciprofloxacin** | **Treatment** | | | |
| --- | --- | --- | --- | --- | --- |
|  | **(μg/mL)** | **0.0 mM Zn** | **0.5 mM Zn** | **1.0 mM Zn** | **Metal cont. water** |
| **Susceptible** | 0.000195313 | 1.045 + 0.060 | 0.785 + 0.029 | 0.575 + 0.051 | 0.775 + 0.006 |
|  | 0.000390625 | 0.991 + 0.013 | 0.757 + 0.013 | 0.604 + 0.086 | 0.775 + 0.008 |
|  | 0.00078125 | 0.991 + 0.041 | 0.768 + 0.040 | 0.588 + 0.051 | 0.750 + 0.015 |
|  | 0.0015625 | 0.987 + 0.018 | 0.745 + 0.033 | 0.560 + 0.027 | 0.762 + 0.004 |
|  | 0.003125 | 0.948 + 0.013 | 0.762 + 0.034 | 0.561 + 0.012 | 0.733 + 0.025 |
|  | 0.00625 | 0.756 + 0.003 | 0.771 + 0.037 | 0.547 + 0.012 | 0.651 + 0.012 |
|  | 0.0125 | 0.496 + 0.012 | 0.683 + 0.012 | 0.517 + 0.021 | 0.418 + 0.008 |
|  | 0.025 | 0.303 + 0.003 | 0.447 + 0.009 | 0.423 + 0.033 | 0.261 + 0.018 |
|  | 0.05 | 0.137 + 0.023 | 0.268 + 0.010 | 0.130 + 0.047 | 0.085 + 0.004 |
|  | 0.1 | 0.071 + 0.002 | 0.103 + 0.008 | 0.071 + 0.007 | 0.068 + 0.001 |
|  | 0.2 | 0.072 + 0.019 | 0.067 + 0.001 | 0.066 + 0.003 | 0.061 + 0.001 |
|  | 0.4 | 0.060 + 0.001 | 0.058 + 0.003 | 0.065 + 0.001 | 0.058 + 0.001 |
| **Resistant** | 0.000195313 | 0.866 + 0.081 | 0.713 + 0.069 | 0.481 + 0.022 | 0.675 + 0.009 |
|  | 0.000390625 | 0.884 + 0.073 | 0.714 + 0.094 | 0.501 + 0.052 | 0.702 + 0.017 |
|  | 0.00078125 | 0.892 + 0.029 | 0.718 + 0.075 | 0.497 + 0.076 | 0.70 + 0.019 |
|  | 0.0015625 | 0.878 + 0.016 | 0.681 + 0.024 | 0.470 + 0.039 | 0.699 + 0.019 |
|  | 0.003125 | 0.878 + 0.017 | 0.683 + 0.018 | 0.469 + 0.027 | 0.692 + 0.013 |
|  | 0.00625 | 0.878 + 0.026 | 0.676 + 0.012 | 0.464 + 0.035 | 0.704 + 0.013 |
|  | 0.0125 | 0.893 + 0.016 | 0.671 + 0.022 | 0.468 + 0.035 | 0.692 + 0.013 |
|  | 0.025 | 0.874 + 0.024 | 0.668 + 0.022 | 0.469 + 0.027 | 0.715 + 0.060 |
|  | 0.05 | 0.822 + 0.008 | 0.648 + 0.018 | 0.460 + 0.025 | 0.679 + 0.012 |
|  | 0.1 | 0.868 + 0.049 | 0.624 + 0.039 | 0.450 + 0.018 | 0.661 + 0.021 |
|  | 0.2 | 0.699 + 0.033 | 0.596 + 0.023 | 0.444 + 0.023 | 0.605 + 0.044 |
|  | 0.4 | 0.519 + 0.022 | 0.536 + 0.061 | 0.450 + 0.026 | 0.530 + 0.022 |

**SI Table 2: Relative growth of the susceptible and the ciprofloxacin resistant strain across a gradient of ciprofloxacin and zinc concentrations.** Growth was quantified based on OD_600_ after 24 h of growth at 37˚C (n=3, average ± SD) compared to the susceptible strain in the absence of antibiotic or metal.

**
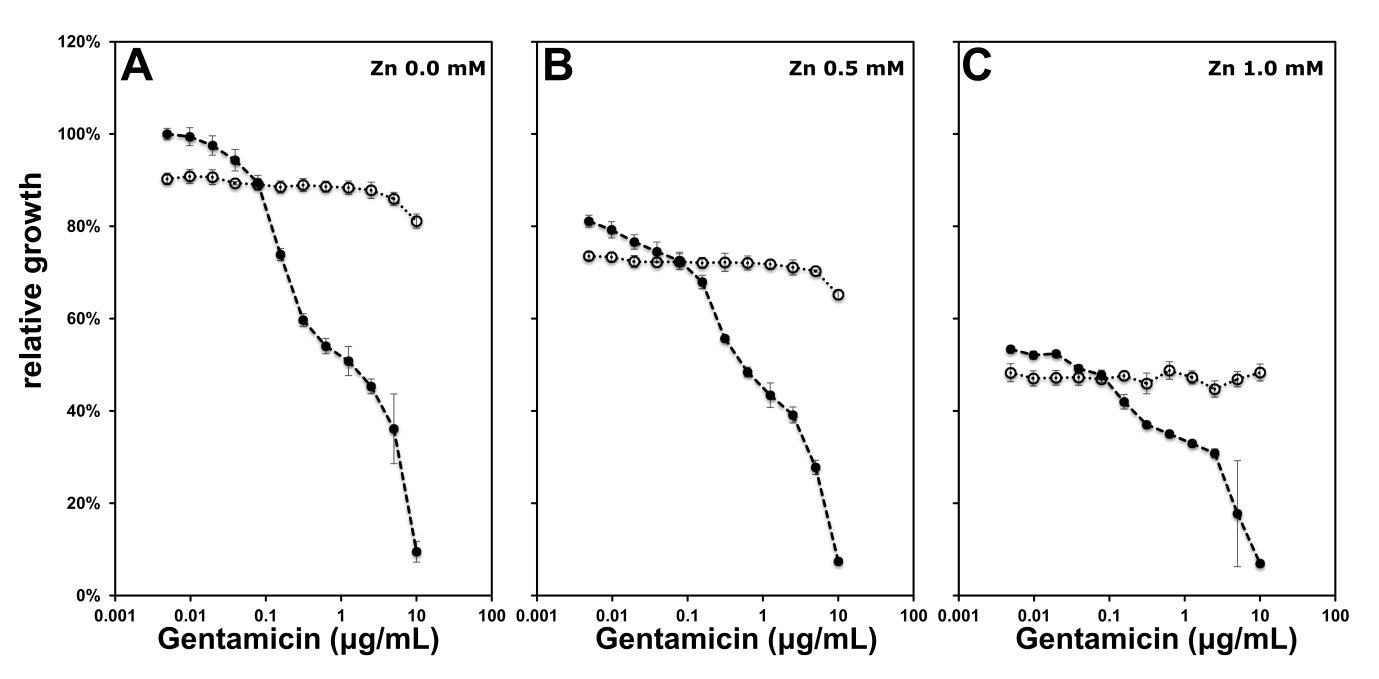
**

**SI Figure 1: Relative growth of the susceptible (filled circles) and the gentamicin resistant (open circles) strain across a gradient of gentamicin and zinc concentrations.** Growth was quantified based on OD_600_ after 24 h of growth at 37˚C (n=3, average ± SD) compared to the susceptible strain in the absence of antibiotic or metal.

**
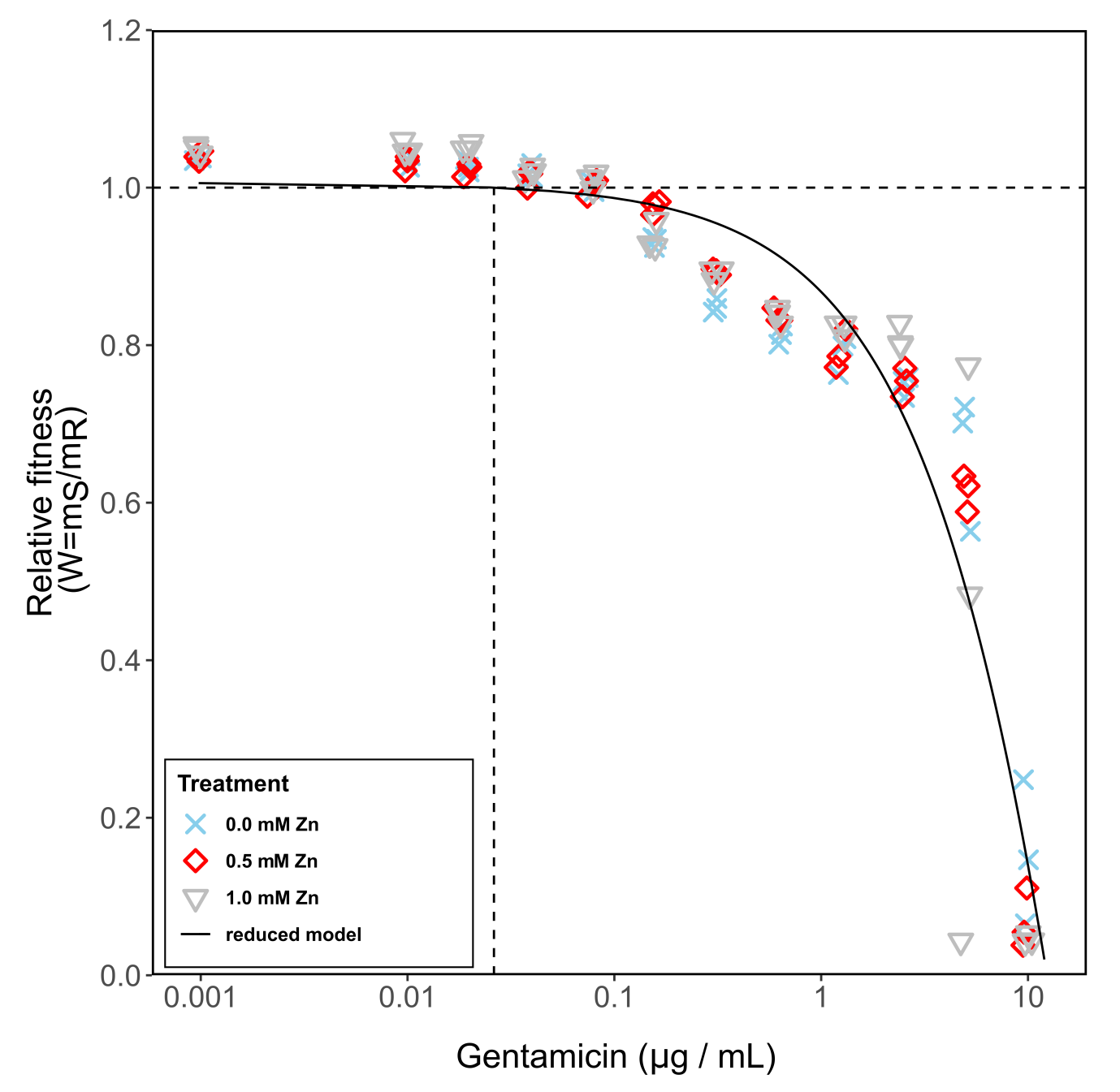
**

**SI Figure 2: Relative fitness of the gentamicin susceptible strain across a gradient of gentamicin and zinc concentrations.** Horizontal dashed line indicates no selection at a relative fitness of *ρ*_s_ = *ρ*_r_ = 1. The intercept with the reduced log-logistic dose response model indicates the minimal selective concentration illustrated with a vertical dashed line. Since no significant influence of Zn on the log-logistic dose response model was detected exclusively the reduced models selection curve with MSC for gentamicin at 0.0242 ± 0.0162 μg/mL is shown.
